# Facial memory ability and self-awareness in patients with temporal lobe epilepsy after anterior temporal lobectomy

**DOI:** 10.1371/journal.pone.0248785

**Published:** 2021-04-01

**Authors:** Hiroaki Hosokawa, Shigenori Kanno, Yoshiyuki Nishio, Iori Kawasaki, Kazumi Hirayama, Atsuko Sunaga, Naotake Shoji, Masaki Iwasaki, Nobukazu Nakasato, Teiji Tominaga, Kyoko Suzuki

**Affiliations:** 1 Department of Behavioral Neurology and Cognitive Neuroscience, Tohoku University Graduate School of Medicine, Sendai, Japan; 2 Department of Rehabilitation, Sendai-Nishitaga National Hospital, Sendai, Japan; 3 Department of Psychiatry and Neurology, Tokyo Metropolitan Matsuzawa Hospital, Setagaya, Japan; 4 Department of Occupational Therapy, Yamagata Prefectural University of Health Sciences, Yamagata, Japan; 5 Department of Psychiatry, National Center Hospital, National Center of Neurology and Psychiatry, Kodaira, Japan; 6 Department of Neurosurgery, National Center Hospital, National Center of Neurology and Psychiatry, Kodaira, Japan; 7 Department of Epileptology, Tohoku University Graduate School of Medicine, Sendai, Japan; 8 Department of Neurosurgery, Tohoku University Graduate School of Medicine, Sendai, Japan; University of Pécs Medical School, HUNGARY

## Abstract

Anterior temporal lobectomy (ATL) is the most common surgical treatment for drug-resistant temporal lobe epilepsy (TLE). Right ATL has been reported to reduce facial memory ability in patients with TLE, as indicated by poor performance on the Warrington Recognition Memory Test for Faces (RMF), which is commonly used to evaluate visual memory in these patients. However, little is known about whether patients with TLE exhibit difficulties in identifying faces in daily life after ATL. The aim of this study was to investigate facial memory ability and self-awareness of face identification difficulties in patients with TLE after ATL. Sixteen patients with TLE after right ATL, 14 patients with TLE after left ATL, and 29 healthy controls were enrolled in this study. We developed the multiview face recognition test (MFRT), which comprises a learning phase (one or three frontal face images without external facial feature information) and a recognition phase (frontal, oblique, or noise-masked face images). Facial memory abilities were examined in all participants using the MFRT and RMF, and self-awareness of difficulties in face identification was evaluated using the 20-item prosopagnosia index (PI20), which has been widely used to assess developmental prosopagnosia. The MFRT performance in patients with TLE after ATL was significantly worse than that in healthy controls regardless of the resected side, whereas the RMF scores in patients with TLE were significantly worse than those in healthy controls only after right ATL. The MFRT performance in patients with TLE after both left and right ATL was more influenced by working memory load than that in healthy controls. The PI20 scores revealed that patients with TLE after left ATL were aware of their difficulties in identifying faces. These findings suggest that patients with TLE not only after right ATL but also after left ATL might have difficulties in face identification.

## Introduction

Epilepsy is one of the most frequent chronic neurological disorders [1–4]. Despite adequate antiepileptic treatment, seizure control is not achieved in approximately 30–40% of patients [5–7]. Anterior temporal lobectomy (ATL) is the most common surgical treatment for drug-resistant temporal lobe epilepsy (TLE) and has been reported to not only provide good seizure control but also to improve the quality of life in patients with TLE and their caregivers [6, 8–12]. However, it is also widely accepted that the left ATL may be accompanied by verbal memory deficits [13–15]. In contrast, the reported visual memory decline in patients with TLE after right ATL has been met with some controversy [16, 17]. One reason for this conflict in visual memory outcomes could be the different measures used to assess visual memory between these studies [18, 19]. However, a meta-analytic review found that the Warrington Recognition Memory Test for Faces (RMF) [20] is the only visual measure that has produced consistent results of reduced facial memory performance in patients with TLE after right ATL [19, 21]. The Warrington Recognition Memory Test comprises word and facial memory tasks. Previous studies have reported a double dissociation between word and facial memory in patients with TLE after left and right ATL [21, 22]. In these studies, word memory ability decreased after left ATL, whereas facial memory ability decreased after right ATL. In addition, several studies have demonstrated that patients with right TLE have poor facial memory performance both before and after right ATL [23–25].

Naturally, facial memory can be influenced by facial perception processing [23]. Previous studies of familiar face recognition and naming have revealed that right TLE patients exhibited presurgical deficits in famous face recognition and postsurgical deficits in both famous face recognition and familiarity judgments without visuoperceptual problems but no significant deficits in naming before and after ATL, whereas left TLE patients demonstrated both presurgical and postsurgical deficits in famous face naming but no apparent deficits in recognition or familiarity judgments [26, 27]. In addition, Drane et al.’s previous study suggested that the deficits in familiar face identification in right TLE patients are derived from a loss of access to semantic information due to the disruption of the ventral visual processing stream [28]. However, Hermann et al. reported that facial recognition performance measured by the Benton Facial Recognition Test [29, 30] declined after both left and right ATL in patients with TLE, although it was not associated with a decline in general intellectual or global and specific measures of visuoperceptual ability [31, 32]. The Benton Facial Recognition Test was developed to detect prosopagnosia, i.e., the inability to recognize faces. The test uses unfamiliar stimuli (unknown faces) that have had all noninternal facial feature information removed, and the subjects indicate which of six oblique face images match the target face image in a frontal view [29, 30]. Although RMF stimuli also use unknown face images, these stimuli contain abundant noninternal facial feature information, such as hairlines and clothes, and are presented in a frontal view [20]. In the recognition phase, one previously presented face image and one distractor face were also concurrently displayed in a frontal view, and the subjects were asked to select the face image that they had seen. Although previous studies have demonstrated that the performance of RMF in TLE patients after left ATL could be preserved, Hermann et al.’s previous studies indicate that TLE patients not only after right ATL but also after left ATL may have difficulties remembering faces with less internal and/or external information if they see the faces at different angles.

In the real world, it is rare to be confronted by so many faces. In addition, people do not always recognize faces from the front, and hairstyles and clothes can change every day. Furthermore, one previous study showed that many participants were able to identify individuals in a modified RMF that removed all internal facial feature information [33]. Although a reduced RMF performance in patients with TLE after right ATL has been demonstrated, it is unclear whether RMF performance can reflect real-world facial identification. In other words, it is not yet known whether patients with TLE have face identification difficulties in daily life after right ATL and whether they have no problem in identifying faces after left ATL. To our knowledge, only a few case studies have reported that patients with TLE hardly identified human faces in daily life after right ATL [34, 35]. Moreover, there have been no group studies of real-world facial memory ability in patients with TLE after ATL. To investigate the facial memory abilities of patients with TLE after ATL in daily life, it seems necessary to use tasks that only deal with internal facial information and more faithfully reproduce real-life face recognition situations.

Several previous studies have reported that patients with focal hippocampal damage due to anoxia or encephalitis have impaired higher-order visuospatial perception and recognition, including unknown faces, when working memory demand is slightly increased [36, 37]. In addition, previous functional MRI studies have demonstrated that the bilateral hippocampus was strongly activated while performing visuospatial working memory tasks with complex spatial stimuli, such as unknown faces and landscapes, and three-dimensional virtual reality rooms, whereas hippocampal activation was not observed during working memory tasks with simple visuospatial stimuli [37, 38]. These studies indicate that the hippocampus plays a crucial role in higher-order visuospatial perception and working memory. Although in these previous studies, only the right or bilateral hippocampi were involved in patients with impaired higher-order visuospatial working memory, deficits in identifying faces in a different view may be more apparent if working memory load increases in TLE patients after left ATL.

The main aims of this study were to investigate facial memory ability and self-awareness of face-identifying difficulties in patients with TLE after ATL. Our hypothesis is that (1) not only right TLE patients but also left TLE patients after ATL exhibit deficits in identifying unknown faces if they see the faces at different angles or the faces are partially hidden; (2) the deficits will become more severe when working memory load increases; and (3) not only right TLE patients but also left TLE patients after ATL are aware of face identification difficulties in their daily life. For these purposes, we developed a new test to assess facial memory that not only uses frontal face images but also oblique face images and those covered with noise. In addition, the test required participants to learn only one or three unknown faces to investigate the alteration of facial memory ability due to working memory load and to better reflect situations encountered in daily life. Moreover, we assessed face identification difficulties in daily life using a validated self-assessment questionnaire. We selected the 20-item prosopagnosia index (PI20), which has been confirmed to be correlated with the Cambridge Face Memory Test scores [39]. It is important to investigate the relationship between subjective complaints of face identification difficulties and actual performance on neuropsychological tests to encourage patients’ social participation and to improve their quality of life.

## Materials and methods

The ethics committees of the Tohoku University Hospital approved this study. Written informed consent was obtained from all participants after they had been given a detailed description of the study.

### Participants

#### Patients with temporal lobe epilepsy

Thirty consecutive patients with drug-resistant TLE who underwent unilateral ATL at Tohoku University Hospital or National Center of Neurology and Psychiatry Hospital were enrolled in this study. The inclusion criteria were as follows: (1) 1 year or more after surgery; (2) ≥ 16 years old; (3) a Performance Intelligence Quotient index on the Wechsler Adults Intelligence Scale-III (WAIS-III) of ≥ 70; (4) a native Japanese speaker; and (5) a best-corrected Snellen acuity ≥ 20/50. The exclusion criteria were as follows: (1) concurrent extratemporal lesions on MRI and (2) no history of neurological or psychiatric disease other than TLE-related conditions.

**Fig 1:**
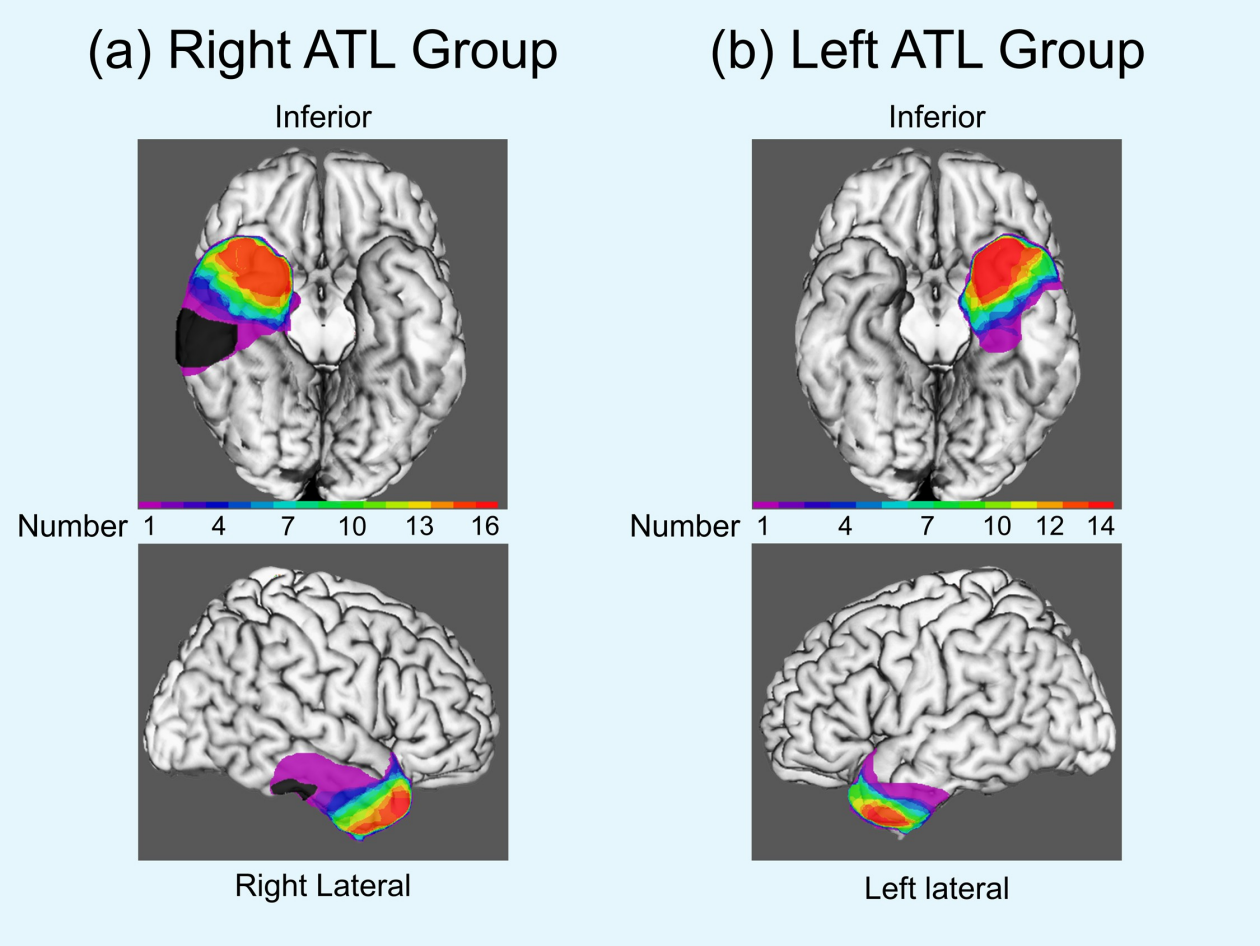
The lateral and basal temporal areas resected in the patients with Anterior Temporal Lobectomy (ATL). The color map indicates the areas resected in the patients with right ATL (a) and the patients with left ATL (b). The colored bar (purple-red) indicates the number of patients with each left or right ATL. A black area indicates the lesion of infarction in one patient with right ATL, which developed immediately after surgery. The structures resected in many patients with anterior temporal lobectomy (green-red) were distributed in the anterior parts of the middle and inferior temporal gyrus, the fusiform gyrus, and the parahippocampal gyrus.

Of the 30 patients, ATL with amygdalohippocampectomy was performed in 24 patients (right: 13 patients; left: 11 patients). The antero-inferior temporal lobe was removed by up to 35 mm on the dominant side and 45 mm on the nondominant side from the tip of the temporal lobe, and then the inferior horn of the lateral ventricle was opened. The amygdala was aspirated subpially to the level of the anterior choroidal artery or optic tract. The mesial and basal temporal structures, including the hippocampus, entorhinal and perirhinal cortex, and anterior part of the parahippocampal and fusiform gyrus, were removed en bloc and further aspirated to the level of the superior colliculus in the anterior–posterior direction. ATL with amygdalectomy was performed in four patients (right: three patients; left: one patient). One of the remaining two patients underwent left ATL with multiple hippocampal transactions, and the other patient underwent left ATL preserving both the amygdala and hippocampus. One patient had a cerebral infarction adjacent to the resected brain area in the right temporal lobe immediately after right ATL. The lateral and basal temporal areas resected in the patients with ATL are demonstrated in Fig 1.

Postoperative seizure outcome was evaluated using Engel’s classification. Of the patients with right ATL (right ATL group), 11 (68.8%) had achieved seizure freedom (Class I), two had rare seizures (Class II), two had a worthwhile improvement (Class III), and one had no change (Class IV) 1 year after surgery. Of the patients with left ATL (left ATL group), 10 (71.4%) had achieved seizure freedom (Class I), two had rare seizures (Class II), one had a worthwhile improvement (Class III), and one had no change (Class IV). The right ATL and left ATL groups did not differ from each other in age at onset, disease duration, or seizure outcome. The demographic data are summarized in Table 1.

**Table 1:** Demographic and neuropsychological characteristics of the participants.

	RATL (n = 16)	LATL (n = 14)	HC (n = 29)	p-value
Age (years)^†^	37.3 (11.0)	33.5 (12.1)	34.6 (7.7)	0.548 ^a^
Sex (female/male) ^‡^	11/5	5/9	16/13	0.192
Handedness (right/left/ambiguous) ^‡^	14/1/1	13/0/1	25/2/2	0.910
Language dominant (left/right/ambiguous/unknown) ^‡^	7/0/4/5	6/1/3/4		0.752
Level of education (years)^†^	13.7 (2.1)	13.6 (2.2)	14.9 (1.4)	0.043^a^
Age of onset (years)^§^	15.0 (10.7)	17.9 (11.8)		0.481
Disease duration (years)^§^	20.9 (15.9)	14.6 (8.8)		0.194
Engel class^‡^	I: 11	I: 10		0.969
	II: 2	II: 2		
	III: 2	III: 1		
	IV: 1	IV: 1		
WAIS-III^§^				
VIQ	86.3 (14.5)	87.3 (12.8)	N/A	0.838
PIQ	88.9 (9.9)	96.7 (12.3)	N/A	0.064
FIQ	86.3 (12.0)	90.4 (12.6)	N/A	0.375
VC	87.3 (13.2)	85.6 (12.5)	N/A	0.724
PO	92.8 (11.4)	97.0 (12.3)	N/A	0.342
WM	89.6 (15.6)	89.6 (18.2)	N/A	0.993
PS	93.2 (10.8)	94.2 (18.9)	N/A	0.854
WMS-R^§^				
Verbal Memory	93.2 (21.4)	81.1 (17.1)	N/A	0.103
Visual Memory	100.7 (10.9)	100.7 (15.7)	N/A	0.996
General Memory	94.5 (18.2)	83.8 (17.6)	N/A	0.114
Attention/Concentration	100.8 (18.8)	93.6 (16.0)	N/A	0.274
Delayed Recall	89.4 (19.3)	83.1 (20.2)	N/A	0.386
Famous face identification task				
Naming^†^/20	13.3 (3.6)	10.4 (5.1)	16.7 (1.7)	<0.001^b^
Recognition^||^/20	20 (17–10)	20 (16–20)	20 (19–20)	0.154
Warrington Recognition Memory Test				
For Words^†^/50	48.6 (1.6)	47.2 (3.2)	49.3 (0.8)	0.005^b^
For Faces^†^/50	35.7 (4.8)	39.1 (3.8)	40.7 (4.4)	0.003^c^

Values are the mean (standard deviation) except for the recognition score of the famous face identification task (median (range)).

FIQ: Full intelligence quotient; HC: healthy controls; LATL: left anterior temporal lobectomy; N/A: not assessed; PIQ: Performance intelligence quotient; PO: Perceptual Organization; PS: Perceptual Speed; RATL: right anterior temporal lobectomy; VC: Verbal Comprehension; VIQ: Verbal intelligence quotient; WAIS-III: Wechsler Adult Intelligence Scale-Third Edition; WM: Working Memory; WMS-R: Wechsler Memory Scale-Revised.

†One-way analysis of variance (ANOVA) and a post hoc Bonferroni test.

‡Chi-Square test.

§Welch’s t-tests.

||Kruskal-Wallis test and a post hoc Mann–Whitney U-test with Bonferroni correction.

^a^Post hoc Bonferroni test revealed that there was no significant difference between the LATL and RATL groups (p = 1.000), between the LATL and HC groups (p = 0.105), or between the RATL and HC groups (p = 0.133).

^b^LATL < RATL = HC.

^c^RATL < LATL = HC

#### Healthy control group

Twenty-nine healthy controls (HCs) matched for age and sex were recruited from the local community through advertisements. Participants with any history of neurological or psychiatric disease and/or impaired visual acuity (a best-corrected Snellen acuity that was poorer than 20/50) were excluded. Although there was no significant difference in handedness among both the ATL groups and the HC group, the averages of education years in patients after left and right ATL groups were significantly lower than those in the HC group (Table 1).

### Neuropsychological assessment

In the present study, all neuropsychological tests, including standardized and developed tests, were administered to the patients after ATL. There was no significant difference in the mean intervals from ATL to assessment between the right and left ATL groups (right ATL group: 15.1 ± 7.0 months, left ATL group: 12.6 ± 1.3 months, p = 0.191 by Student’s t-test). All patients underwent the following standardized neuropsychological tests: the WAIS-III for verbal capacities, visuoconstructive functions, working memory, and cognitive speed [40]; the Wechsler Memory Scale-Revised (WMS-R) for verbal and visual memory functioning [41]; and the Warrington Recognition Memory Test for facial and word memory abilities [20]. To eliminate language influence on the stimuli, the English words in the original Warrington Recognition Memory Test for words were replaced with Japanese words. Additionally, the Caucasian faces in the original RMF were replaced with Japanese faces to eliminate racial influences on stimuli recognition.

In addition, to evaluate retrograde facial memory and familiar face recognition ability, we developed a famous face identification task in which participants were presented with 20 faces of Japanese actors, athletes, or politicians (10 male and 10 female faces). Participants were required to provide both the first and last names of the famous individuals. If they could not correctly name the famous person, they were asked to provide the information about the famous person. These tests yield two scores: the naming score, which is the number of total correct responses (a participant correctly answers both the first and last names of the famous individual), and the recognition score, which is calculated by adding the number of total correct responses to the number of total correct but insufficient responses (a participant correctly answers either the first or last name or correctly provides one or more pieces of information about the famous person). Each score ranges from 0 to 20. The famous face identification task was administered to all participants.

### Multiview face recognition test

#### Stimuli

We collected 144 nonfamous Japanese faces (72 female) for test stimuli, of which 60 were drawn from the face database of the Advanced Telecommunications Research Institute International Inc. and the others were obtained from local volunteers. A 10-point rating of facial emotional valence was assessed in each face image by 10 healthy volunteer raters (5 women; mean age, 31.7 ± 1.9 years). Sixty neutral face images were selected in order from the average emotional valences closest to 5.5 points. The average emotional valence of the selected male and female face images was 4.70 (SD = 0.40) points and 4.89 (SD = 0.59) points, respectively. There was no significant difference in valence between the male and female face images (p = 0.150). Hair-removed frontal and oblique [left—30 degrees] views of each face were generated using FaceGen software (Singular Inversion). We removed the facial blemishes manually using Adobe Photoshop CS 6.0 and Adobe Illustrator CS 5.1. All face images were transformed into grayscale with a black background. Noise-masked face images were created by overlaying black-and-white visual noise of spatial frequencies of 1/f^3^ on frontal face images. Selected examples of face stimuli are shown in Fig 1.

#### Procedure

**Fig 2:**
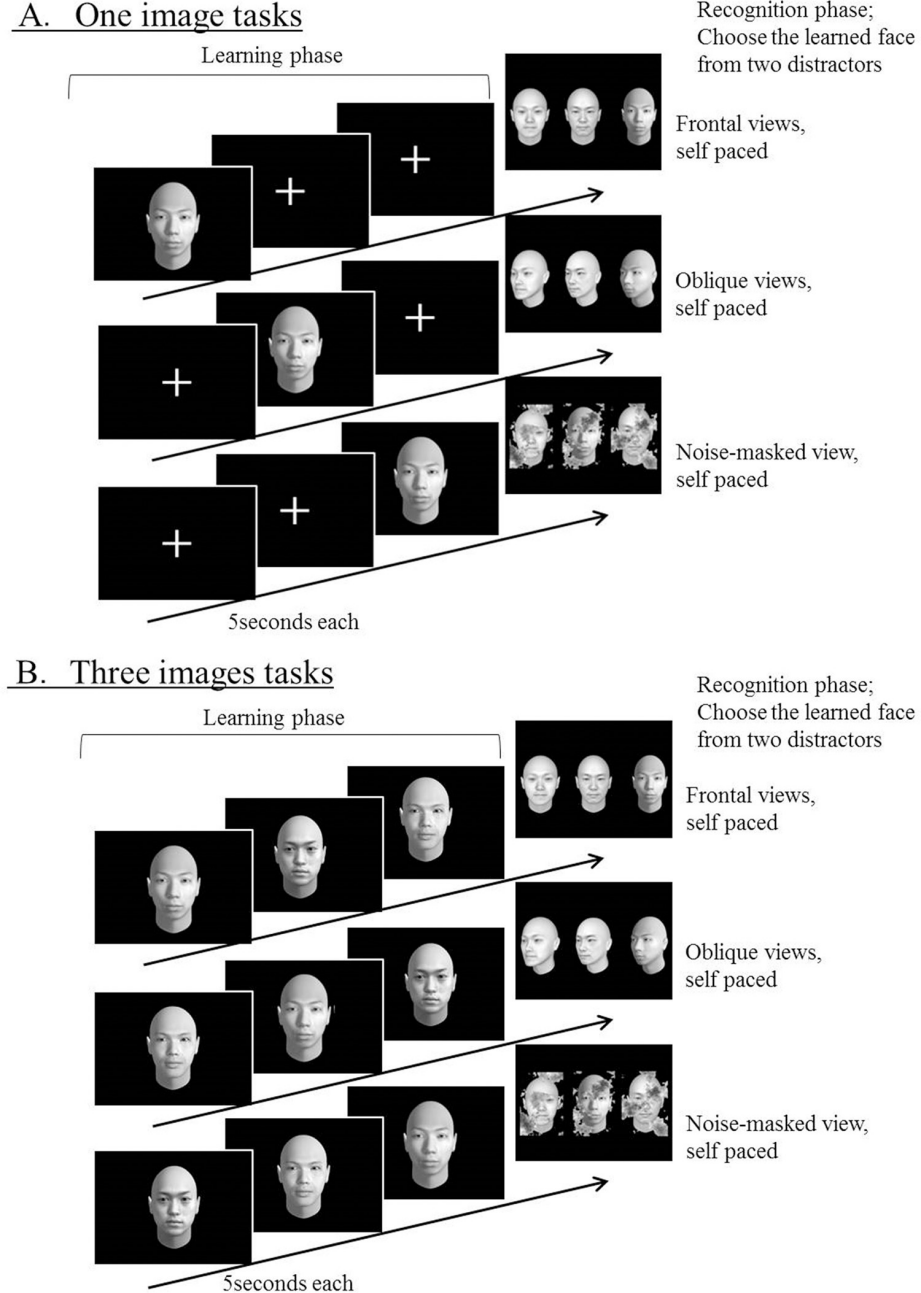
Procedure of the multiview face recognition test. Participants were shown one (A) or three (B) nonfamous Japanese faces in consecutive random order for 5 s. Immediately following this, participants were presented with three forced-choice items consisting of a target face and two distractor faces. Participants were required to select the face that they had seen before.

Participants sat in front of a personal laptop computer (Lenovo B590, 15.6-inch display). The computer was placed in an easily viewable and operable position. The participants were instructed both verbally and in written instructions. Each trial of the test consisted of a learning phase and a recognition phase. During the learning phase, participants were asked to remember frontal face images that were displayed on a screen for 5 s. This presentation time and the angle of face (oblique face image) was defined based on a pilot study in 7 healthy volunteer subjects (4 women; mean age, 32.3 ± 2.4) so that the total accuracy rate of the MFRT became approximately 80%. We varied the numbers of frontal face images displayed (one or three images) (Fig 1A and 1B). In the one face image tasks, two crosshair images were presented instead of face images. Because the order of displaying the target face image needed to be randomized, the time interval between the learning and recognizing the target face was accordingly varied (0, 5, or 10 s). In the recognition phase, one previously presented face image and two distractor faces were concurrently displayed. These images were frontal faces, oblique faces, or noise-masked faces. Participants were asked to select the face that they had seen before by pressing a button. There was no time restriction for responses, and no feedback was provided. This procedure was repeated for frontal, oblique, and noise-masked face images. The test comprised six blocks (two levels of working memory load (1 or 3 face images) × three types of face images (frontal, oblique, or noise-masked). Each block had 12 trials. The order of trials was randomized across blocks, and the order of blocks was also randomized across participants. Fig 2 shows a summary of the MFRT. The MFRT was administered to all participants.

#### Scoring

We found significant differences in the scores between each time interval between the learning and recognition phases (0, 5, or 10 s), both in the one-image and three-image blocks in the HC group (S1 Table). As shown by one-way analysis of variance (ANOVA) and a post hoc Bonferroni test in the HC group, the mean recognition score after 10 s was significantly lower than those at 0- and 5-s intervals in the one-image blocks (F (2, 27) = 7.51, p = 0.003, η2 = 0.357), whereas the mean recognition scores after 5- and 10-s intervals were significantly lower than those at the 0-s interval in the three-image block (F (2, 27) = 17.82, p < 0.001, η2 = 0.569). To correct the difficulties in recognizing faces arising from time intervals between the learning and recognition phases, the scores derived from each time interval were weighted according to the average number of correct answers in the HC group. The scores were defined as follows: (1) 1.00 point (with no time interval both in the one-image and three-image blocks and after the 5-s interval in the one-image blocks); (2) 1.09 points (after 10 s in the one-image blocks); and (3) 1.21 points (for the 5- and 10-s intervals in the three-image blocks). The score of each block, which is calculated by dividing the total points of each block by the possible highest points of each block (percent), was used for statistical analyses.

### Self-reported measure of facial recognition

To assess patients’ insight into their own facial recognition abilities, we used the PI20 [39], which is a self-report instrument that has been widely used to assess developmental prosopagnosic traits. The PI20 comprises 20 questions that assess the difficulty of face recognition and memory in daily life. Each item is rated on a 5-point Likert scale (from strongly agree to strongly disagree). Fifteen items are scored positively (whereby strongly agree is scored as 5 and strongly disagree is scored as 1), and five items are reverse-scored (strongly agree is scored as 1 and strongly disagree is scored as 5). The total PI20 score ranges from 20 to 100 points, whereby a higher score reflects stronger feelings of difficulty in face recognition. We could not perform the PI20 in three patients with right ATL and one patient with left ATL.

### Statistical analyses

One-way ANOVA and post hoc Bonferroni tests were used for between-group comparisons in the Warrington Recognition Memory Test scores (words and faces), and Welch’s t-test was used to compare the other neuropsychological test scores, except for the recognition score of the famous face identification task and the MFRT scores, between the left and right ATL groups. A three-way repeated measures ANOVA was used to analyze between-group differences (Group: left ATL, right ATL, or HC) in the MFRT scores for the three stimulus conditions (View: frontal view, oblique view, or noise-masked view) and between the two working memory loads (Memory: one image or three images). The Kruskal–Wallis test and a post hoc Mann–Whitney U-test with Bonferroni correction were used to compare the recognition score of the famous face identification task and PI20 scores between the three groups.

Statistical analyses were performed using IBM SPSS statistics software (version 26.00; IBM SPSS Inc., Armonk, NY, USA), and the threshold for statistical significance was set at p < 0.05.

## Results

### Standard neuropsychological assessments

The neuropsychological assessment results are summarized in Table 1. Although the left and right ATL groups did not differ from each other in any index of the WAIS-III and WMS-R score, the mean intelligence quotient index of the WAIS-III in the right ATL group tended to be lower than that in the left ATL group. The mean naming score of the famous face identification task was significantly different among the three groups (F (2, 56) = 18.35, p = 0.001, η^2^ = 0.396). Post hoc pairwise comparisons with Bonferroni correction revealed that the mean naming score of the famous face identification task score in the left ATL group was significantly lower than that in the right ATL (p = 0.050) and HC groups (p < 0.001). In contrast, there were no significant differences in the median recognition scores of the famous face identification task among the three groups (H = 3.74, df = 2, p = 0.154). The mean scores of the Warrington Recognition Memory Test for words (F (2, 56) = 5.87, p = 0.005, η^2^ = 0.173) and for faces (F (2, 56) = 6.67, p = 0.003, η^2^ = 0.192) were significantly different among the three groups. Post hoc pairwise comparisons with Bonferroni correction revealed that the Warrington Recognition Memory Test for words score in the left ATL group was significantly lower than that in the HC group (p = 0.003), whereas there were no such significant differences between the right ATL and HC groups (p = 0.791) or between the left and right ATL groups (p = 0.126). In contrast, the RMF score in the right ATL group was significantly lower than that in the HC group (p = 0.002), and there were no such significant differences between the left ATL and HC groups (p = 1.000) or between the left and right ATL groups (p = 0.105).

### The results of the MFRT

**Fig 3:**
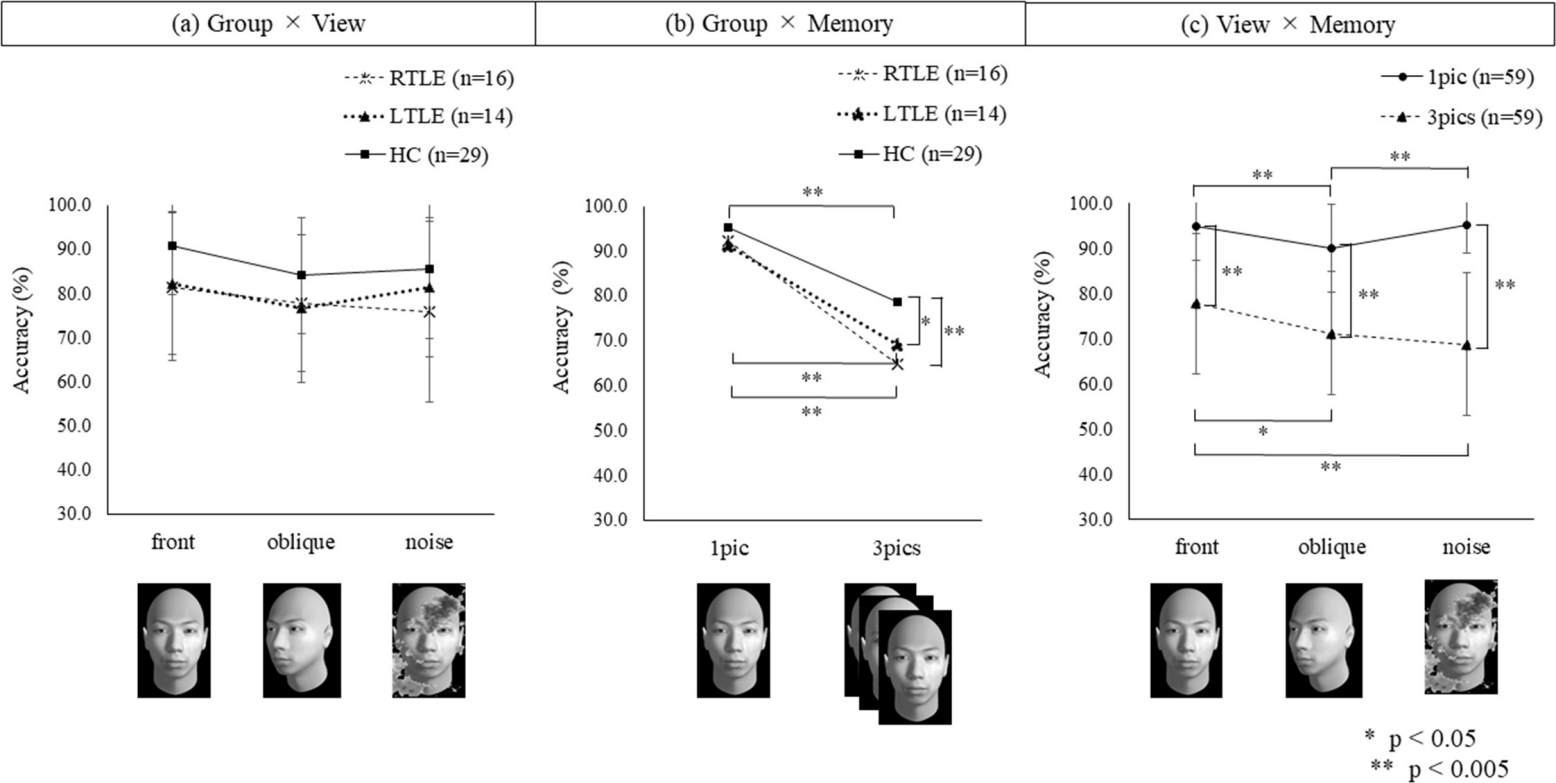
Results of the multiview face recognition test. (a) Group (patients with temporal lobe epilepsy after right anterior temporal lobectomy, after left anterior temporal lobectomy, or healthy controls) and View (frontal view, oblique view, or noise-masked view). (b) Group and Memory (one image or three images). (c) View and Memory. *p < 0.05. **p < 0.005.

The MFRT results are summarized in Table 2, and Fig 3 provides a summary of the results of a three-way repeated measures ANOVA in MFRT scores. There was no significant second-order interaction among Group, View, and Memory (F (4, 112) = 0.93, p = 0.449, η^2^ = 0.032) for the MFRT scores. However, the interactions between Group and Memory (F (2, 56) = 7.54, p = 0.001, η^2^ = 0.212) and between View and Memory (F (2, 55) = 10.15, p < 0.001, η^2^ = 0.270) were significant. There was no significant interaction between Group and View (F (4, 112) = 1.17, p = 0.153, η^2^ = 0.058). The main effects of Group (F (2, 56) = 6.66, p = 0.003, η^2^ = 0.192), View (F (2, 55) = 14.66, p < 0.001, η^2^ = 0.348), and Memory (F (1, 56) = 307.91, p < 0.001, η^2^ = 0.844) were significant. According to post hoc pairwise comparisons with Bonferroni correction, the mean MFRT scores of the left and right ATL groups were significantly worse than those of the HC group (left ATL group versus HC group: p = 0.031; right ATL group versus HC group: p = 0.006). There was no significant difference in the mean MFRT scores between the left and right ATL groups (p = 1.000). In addition, the mean scores for oblique view and noise-masked view trials were significantly worse than those for the frontal view trial (oblique view versus frontal view: p < 0.001; noise-masked view versus frontal view: p = 0.001). There was no significant difference in the mean MFRT score between oblique view and noise-masked view trials (p = 0.852). Moreover, the mean MFRT score in the three-image tasks was significantly lower than that in the one-image tasks (p < 0.001).

**Table 2:** Results of the multiview face recognition test.

Group	Views	1 picture	3 pictures
		Scores %	Scores %
RATL	Front	94.1 (9.1)	71.6 (14.1)
	Oblique	90.9 (8.5)	65.8 (13.2)
	Noise	93.6 (5.8)	58.2 (13.9)
LATL	Front	92.7 (9.3)	72.1 (16.0)
	Oblique	87.2 (13.3)	66.3 (13.7)
	Noise	93.3 (6.8)	69.5 (13.6)
HC	Front	96.8 (5.3)	85.0 (12.5)
	Oblique	92.0 (8.0)	76.3 (13.0)
	Noise	96.7 (5.8)	74.4 (15.0)

Data are given as the mean (standard deviation).

HC: healthy controls; LATL: left anterior temporal lobectomy; RATL: right anterior temporal lobectomy.

In the interaction between Group and Memory, there was no significant simple main effect of Group in the one-image tasks (F (2, 56) = 1.97, p = 0.149, η^2^ = 0.066), whereas the simple main effect of Group in the three-image tasks (F (2, 56) = 8.62, p < 0.001, η^2^ = 0.235) was significant. In contrast, the simple main effect of Memory was significant in each group (left ATL group: F (1, 56) = 77.52, p < 0.001, η^2^ = 0.581; right ATL group: F (1, 56) = 142.97, p < 0.001, η^2^ = 0.720; HC group: F (1, 56) = 95.27, p < 0.001, η^2^ = 0.624). Post hoc Bonferroni contrast analysis tests revealed that the mean MFRT scores in the left and right ATL groups were significantly worse than those in the HC group on the three-image tasks (left ATL group versus HC group: p = 0.035; right ATL group versus HC group: p = 0.001). There was no significant difference in the mean MFRT scores between the left and right ATL groups on the three-image tasks (p = 0.927). In addition, the mean MFRT score on the three-image tasks was significantly worse than that on the one-image tasks in each group (left ATL group: p < 0.001; right ATL group: p < 0.001; HC group: p < 0.001).

In the interaction between View and Memory, simple main effects of View in both the one-image and three-image tasks were significant (one-image tasks: F (2, 55) = 11.31, p < 0.001, η^2^ = 0.291; three-image tasks: F (2, 55) = 11.12, p < 0.001, η^2^ = 0.288). Furthermore, simple main effects of Memory were also significant for each view (frontal view: F (1, 56) = 99.39, p < 0.001, η^2^ = 0.640; oblique view: F (1, 56) = 126.23, p < 0.001, η^2^ = 0.693; noise-masked view: F (1, 56) = 223.72, p < 0.001, η^2^ = 0.800). Post hoc Bonferroni contrast analysis tests revealed that the mean MFRT score for the oblique view was significantly worse than those for the frontal view and noise-masked view on the one-image tasks (frontal view versus oblique view: p < 0.001; oblique view versus noise-masked view: p < 0.001). In addition, the mean MFRT scores for the oblique view and noise-masked view were significantly worse than those for the frontal view on the three-image tasks (frontal view versus oblique view: p = 0.005; frontal view versus noise-masked view: p < 0.001). There was no significant difference in mean MFRT scores between the oblique view and noise-masked view on the three-image tasks (p = 0.868). Moreover, the mean MFRT score on the three-image tasks was significantly worse than that on the one-image tasks for each view (frontal view: p < 0.001; oblique view: p < 0.001; noise-masked view: p < 0.001).

### The PI20 results

The PI20 results are shown in Table 3. For the Kruskal–Wallis test, the PI20 scores were significantly different among the three groups (H = 6.50, p = 0.04). The Mann–Whitney U-test with Bonferroni correction (significance was defined for p-values < 0.05/3) revealed that the PI20 score in the left ATL group was significantly higher than that in the HC group (p = 0.01). There was no significant difference in the PI20 score between the right ATL and HC groups (p = 0.45).

**Table 3:** The 20-item prosopagnosia index results.

	RATL (n = 13)		LATL (n = 13)		HC (n = 29)		p-value		
	Median	Range	Median	Range	Median	Range	RATL vs. LATL	RATL vs. HC	LATL vs. HC
PI20	43	23–74	53	27–69	40	20–65	0.695^a^	0.453^a^	0.011^a^

RATL: right anterior temporal lobectomy; LATL: left anterior temporal lobectomy; HC: healthy control; PI20: the 20-item prosopagnosia index; SD: standard deviation.

^a^Post hoc Mann–Whitney U-test with Bonferroni correction. The significance was defined for p-values < 0.05/3.

## Discussion

We investigated facial memory ability and self-awareness of face identification difficulties in patients with TLE after ATL using MFRT and PI20. The major findings were as follows: (1) the MFRT performance in patients with TLE after ATL was significantly worse than that in HCs regardless of the resected side; and (2) the PI20 score was higher in patients with TLE after left ATL but was not higher in patients with TLE after right ATL than in HCs.

### Patients with TLE after left ATL

As previous studies have demonstrated, RMF performance was normal in TLE patients after left ATL in the current study [21, 22]. On the other hand, the TLE patients exhibited poorer MFRT performance after both left and right ATL than the HC participants. These results were consistent with our expectations. However, it cannot be said that the reason for the poor MFRT performance in the TLE patients after left ATL entirely corresponds with our hypothesis. Although the TLE patients had difficulties remembering faces with less internal and/or external information after left ATL if they saw the face from different views or the faces that were partially hidden, these difficulties were also observed in the HC participants, and there was no significant interaction between the participants’ groups and views. Therefore, the poor MFRT performance in the TLE patients after left ATL was not mainly caused by remembering faces from different angles or partially hidden faces.

The discrepancy between the RMF and MFRT results in the TLE patients after left ATL is likely to have arisen from differences between the two tests. The RMF stimuli contained abundant noninternal facial feature information, such as hairline and clothes, whereas the MFRT stimuli were hair-removed. Given that the face images used in the MFRT contain less nonfacial feature information, the clues for learning and recognizing face images were more limited than in the RMF. In addition, the MFRT requires participants to recognize face images that are different from the learned face images. We suggest that this requires the ability to complement or compensate for incomplete face images. However, the TLE patients after left ATL exhibited good facial memory performance, despite the less informative face images on the one-image tasks. The poor performance on the MFRT in the TLE patients after left ATL only became obvious on the three-image tasks. These findings indicate that difficulties in learning or remembering less informative face images in TLE patients after left ATL were accompanied by an increased working memory load, which is coincident with one of our hypotheses.

Previous studies of patients with focal hippocampal damage due to anoxia or encephalitis, amnestic mild cognitive impairment, or Alzheimer’s disease and functional MRI studies have indicated that the hippocampus plays a crucial role in higher-order visuospatial working memory [36–38, 42, 43]. In the current study, the left hippocampus was involved in almost all patients with TLE who received left ATL, although the patients with impaired-high-order visuospatial working memory in these previous studies had only right or bilateral hippocampal damage. Thus, the present study suggested that left hippocampal resection may cause impaired higher-order visuospatial working memory in patients with left TLE. One possible reason for this finding is that the process of complementing or inferring from incomplete face image information and its associated working memory maintenance requires complementary bilateral temporal lobe activity. A previous magnetic resonance spectroscopic imaging study reported that 50% of patients with TLE with unilateral electroencephalographical and MRI abnormalities showed a decreased N-acetylaspartate concentration in the contralateral hippocampus, which could correctly predict unsuccessful surgical outcomes [44]. This finding suggests that patients with TLE may have bilateral hippocampal dysfunction. If so, higher-order visuospatial working memory could be easily impaired by ATL of either hemisphere.

In addition, a previous study reported that patients with medial temporal lobe lesions that were involved in not only the hippocampus but also the perirhinal cortex, which was involved in almost all patients after ATL in the present study, showed impaired discrimination of unfamiliar faces presented from differing viewpoints [45]. Moreover, previous functional MRI studies of healthy participants demonstrated that the perirhinal cortex and posterior hippocampus were more active for discriminations involving novel stimuli, such as unknown faces, scenes, and objects, presented from different viewpoints [46]. Apart from our results, these previous studies indicated that poor performance in the MFRT in TLE patients after ATL might be associated with deficits in discriminating unfamiliar faces presented from differing viewpoints. There was a significant interaction between working memory load and views in our study, although this finding was not specific to TLE patients. The poor performance on the MFRT in TLE patients after left ATL due to working memory load might be indirectly emphasized by discriminating unfamiliar faces from different viewpoints.

The PI20 scores in patients with TLE after left ATL were higher than those in the HC group. This finding could not be predicted from the results of the RMF, since the RMF scores in patients with TLE after left ATL were not different from those of HCs in both the current and previous studies [21, 22]. We suggest that in daily life, patients with TLE after left ATL might have difficulties in face identification for faces seen from a different viewpoint or that are partially hidden. Moreover, the naming scores of the famous face identification tasks in TLE patients after left ATL were significantly poorer than those in TLE patients after right ATL and HC participants in the current study. In contrast, the recognition performance of the famous face identification tasks in TLE patients after left ATL was equivalent to those in TLE patients after right ATL and HC participants. These findings are coincident with previous studies of famous face naming and support the theory that the left anterior temporal lobe plays a role in linking semantic information to the language system to produce a specific name [26–28].

### Patients with TLE after right ATL

In the present study, the performance of the patients with TLE after right ATL on both the RMF and MFRT was significantly lower than that of the HC participants. This finding is coincident with previous studies and our hypothesis [21–25]. It is suggested that not only immediate facial (or nonverbal) memory but also facial (or higher-order visuospatial) working memory are impaired in TLE patients after right ATL. On the other hand, the naming and recognition scores of the famous face identification task in TLE patients after right ATL were equivalent to those of HC participants in our study. Previous studies have reported that after right ATL, patients exhibited poor face recognition performance on famous face identification tasks despite no visuoperceptual or visuospatial abnormalities [18, 19, 26–28]. In addition, the area of the resected anterior temporal lobe in our study was almost the same as that in these previous studies. One possible reason for this discrepancy between our study and these previous studies in the famous face identification tasks is a ceiling effect for the recognition scores of the famous face identification task in our study. In the HC participants, although the result of the RMF we created was equivalent to the English version of the RMF in HC participants [20], the accuracy of face recognition on the famous face identification tasks we created was obviously higher than that in the previous studies. Therefore, it was possible that we could not detect deficits in famous face recognition in TLE after right ATL. In addition, because we did not administer the Benton Facial Recognition Test, we could not compare the severity of deficits in unfamiliar face recognition ability among our study and these previous studies. Ultimately and unfortunately, our findings could not conclude whether the lower RMF and MFRT performance in TLE patients after right ATL was associated with facial visuoperceptual or visuospatial impairment.

Although there was no significant difference in the mean visual memory index of the WMS-R between the TLE patients after right ATL and after left ATL, the mean intelligence quotient index of the WAIS-III in the TLE patients after right ATL tended to be lower than that after left ATL. The mean level of performance intelligence in the TLE patients after right ATL was not abnormal. However, it was possible that the performance on the MFRT in these patients was influenced by their intelligence.

Surprisingly, the PI20 scores in the patients with TLE after right ATL were not higher than those in the HC participants. The TLE patients after right ATL did not seem to be aware of face identification impairment in their daily life, despite their significantly reduced objective unfamiliar face identification task performance. One possible explanation for this finding is that patients with TLE after right TLE might have anosognosia for visual memory impairment. Previous studies have demonstrated that very few TLE patients are aware of their significant memory decline after right ATL [47, 48]. Sawrie et al. reported that only 6.9% of patients who underwent right ATL complained of memory decline, despite a high rate of memory impairments on objective measures. Thus, previous studies have suggested that subjective memory decline after ATL is not necessarily consistent with objective memory decline. Anosognosia is common in patients with right hemisphere lesions [49, 50]. A previous neuroimaging study in patients with traumatic brain injury showed that overestimation of their postinjury level of sociocognitive function was associated with hypometabolism in the right prefrontal cortex and right temporal pole [51]. Because the right temporal pole was removed in all patients with TLE in the current study, the TLE patients exhibited underestimation of memory decline after right ATL. Another possible reason is that the patients with TLE in the current study might have a high rate of face identification improvement after right TLE. Several previous studies have reported that some patients who underwent right ATL experienced improvements in objective visual memory performance [15, 52, 53]. However, we could not determine whether facial memory ability was improved by right ATL because we did not implement the MFRT, RMF, or PI20 before surgery.

### Limitations and future directions

Our study has several limitations. First, we did not validate the developed neuropsychological tests, including the MFRT, famous face identification task, and Japanese version of RMF. We did not evaluate test-retest reliability for these neuropsychological tests. Therefore, we could not conclude whether the differences in performance on these neuropsychological tests among the TLE patients and HC participants were evident. In addition, as mentioned above, the recognition scores of the famous face identification task we created demonstrated a ceiling effect in the TLE patients and HC participants. Unfortunately, it is highly likely that we could not reliably detect deficits in famous face recognition in patients after right ATL.

Second, we did not implement neuropsychological tests, including the MFRT and PI20, before surgery. Thus, we could not investigate whether the MFRT performance in the patients with TLE was reduced due to ATL or whether stronger awareness of difficulties in face recognition in daily life was derived from a left ATL.

Third, we only evaluated facial memory ability. However, in patients with hippocampal damage, impaired higher-order visuospatial perception or recognition has been observed when working memory demand is increased, even though face stimuli were not used [37–40]. Therefore, we could not elucidate whether the facial memory ability measured using the MFRT was specific to faces or to complex visuospatial stimuli.

Finally, some TLE patients did not undergo PI20 before either or both MFRT and RMF were implemented. Therefore, the results of MFRT and/or RMF could affect the PI20 scores in these patients. Moreover, the PI20 score is influenced by various factors, including memory function and face perception. The daily demand for face recognition in social life may be altered by occupation and/or household [54]. Environments in which interpersonal relationships are important, such as customer service, sales staff, teachers, and students, require better face recognition ability. Therefore, people working in such environments may be more aware of their face recognition dysfunction. However, we did not consider the participants’ occupation. Moreover, we did not examine the subjective severity of affective depression. Several previous studies have reported that patients with TLE are frequently depressed and anxious, particularly those with left-sided TLE [55]. Thus, the higher PI20 scores in the patients with TLE after left ATL in the current study might be associated with the severity of depression or anxiety.

In conclusion, our results suggest that the ability to remember faces with less internal and/or external information is impaired when visuospatial working memory load increases in patients with TLE who underwent ATL, regardless of the resected side. However, validation of the MFRT is necessary to confirm our findings. In the future, we aim to investigate memory of not only unfamiliar but also familiar faces and related multiaspect information about patients with TLE before and after ATL to elucidate whether ATL affects facial memory ability, which could help improve the quality of life and surgical outcome of ATL in patients with TLE.

## Supporting information

S1 Table The average number of correct answers for the MFRT for each time interval between the learning and recognition phases in the HC group. (DOCX)

S2 Table The demographics of each participant. (PDF)

S3 Table The MFRT results of each participant. (PDF)
