# Supplementary material for: Facial memory ability and self-awareness in patients with temporal lobe epilepsy after anterior temporal lobectomy
Source: PLoS One. 2021 Apr 1;16(4):e0248785. doi: 10.1371/journal.pone.0248785 (PMC8016293; doi:10.1371/journal.pone.0248785)
Supplement: S1 Table — (DOCX) [file pone.0248785.s001.docx]

**S1 Table**

The average number of correct answers for the MFRT for each time interval between the learning and recognition phases in the HC group (n = 29).

| Tasks | 0 s | 5 s | 10 s | p-Value | | |
| --- | --- | --- | --- | --- | --- | --- |
|  |  |  |  | 0 s vs 5 s | 0 s vs 10 s | 5 s vs 10 s |
| One-image tasks | 11.8 (0.5) | 11.7 (0.5) | 10.8 (1.4) | 1.000^a^ | 0.002^a^ | 0.002^a^ |
| Three-image tasks | 10.7 (0.9) | 9.4 (1.8) | 8.4 (2.3) | 0.001^a^ | < 0.001^a^ | 0.060^a^ |

Data are given as the mean (standard deviation). p-values of pairwise time interval comparisons.

MFRT: Multi-view face recognition test; HC: Healthy control; SD: Standard deviation.

^a^Post-hoc Bonferroni test.
