# Supplementary material for: Facial memory ability and self-awareness in patients with temporal lobe epilepsy after anterior temporal lobectomy
Source: PLoS One. 2021 Apr 1;16(4):e0248785. doi: 10.1371/journal.pone.0248785 (PMC8016293; doi:10.1371/journal.pone.0248785)
Supplement: S2 Table — (PDF) [file pone.0248785.s002.pdf]

S2 Table. **The demographics of each participant.**

Subject	Age	Sex	Handedness	Language dominant	Level of education (year)	Age of onset	Disease duration (year)	Engel class	Interval from ATL to assessments (month)	Resection				WAIS-III							WMS-R					Famous face identification task		Warrington Recognition Memory Test		PI20 (/100)
										ATL	Amygdala	Hippocampus	Others	VIQ	PIQ	FIQ	VC	PO	WM	PS	Verbal Memory	Visual Memory	General Memory	Attention/ Concentration	Delayed Recall	Naming (/20)	Recognition (/20)	For Words (/50)	For Faces (/50)	
RATL1	33	F	Right	Unknown	16	29	1	I	41	+	+	+		111	90	102	104	101	113	97	133	106	130	138	126	18	20	50	40	N/A
RATL2	28	F	Right	Left	13	13	14	I	13	+	+	+		60	76	64	61	85	67	78	100	79	93	60	87	12	20	49	35	N/A
RATL3	30	F	Right	Left	12	3	23	II	18	+	+	+		96	99	97	93	103	103	105	96	104	97	121	107	18	20	49	41	33
RATL4	54	F	Right	Left	12	8	45	I	13	+	+	+		88	87	86	97	87	83	86	122	96	116	110	104	11	17	46	33	57
RATL5	19	F	Right	Left	12	9	9	I	13	+	+	+		75	79	74	82	99	74	81	98	85	94	80	69	7	20	50	41	55
RATL6	43	F	Right	Ambiguous	11	18	24	IV	13	+	+	+		71	94	80	71	99	69	89	71	101	77	83	79	10	20	50	41	43
RATL7	44	F	Right	Unknown	14	7	35	III	13	+	+	+		91	92	91	80	97	102	81	59	111	70	112	78	10	18	49	35	74
RATL8	51	M	Right	Unknown	12	11	39	I	13	+	+	+		75	98	84	86	103	67	81	74	102	80	94	95	10	20	45	38	50
RATL9	39	F	Right	Unknown	12	3	35	III	13	+	+	-		76	90	81	73	99	96	94	78	108	85	110	91	13	20	48	34	35
RATL10	31	F	Right	Left	16	25	6	I	13	+	+	-		85	90	86	86	89	88	100	90	114	96	103	100	18	20	50	34	35
RATL11	53	M	Right	Left	18	7	45	I	13	+	+	+	CI	107	88	99	104	97	109	100	98	108	102	102	106	13	20	47	31	34
RATL12	42	F	Right	Ambiguous	12	9	32	I	13	+	+	+		80	72	74	84	72	81	105	79	101	83	77	68	11	20	50	45	N/A
RATL13	21	F	Right	Unknown	13	15	5	I	13	+	+	+		71	74	70	76	72	94	92	76	87	74	103	64	17	20	47	33	57
RATL14	36	M	Left	Left	16	18	17	I	13	+	+	+		99	99	99	93	101	111	84	102	108	104	110	75	15	20	48	32	23
RATL15	46	M	Right	Ambiguous	14	43	2	II	13	+	+	-		99	108	103	104	106	94	105	131	114	129	110	117	12	20	50	30	36
RATL16	26	M	Ambiguous	Ambiguous	16	22	3	I	14	+	+	+		96	86	91	102	75	83	113	84	87	82	100	65	18	20	50	28	72
LATL1	27	M	Right	Ambiguous	14	24	2	I	13	+	+	+		66	82	70	66	81	69	100	65	77	63	84	56	7	20	38	37	N/A
LATL2	22	M	Right	Left	10	10	11	I	13	+	+	MST		77	91	82	71	101	111	92	70	111	76	104	75	14	20	47	40	39
LATL3	45	M	Right	Left	15	25	19	I	12	+	+	+		106	106	107	93	97	128	110	85	92	85	128	77	10	20	48	37	62
LATL4	20	M	Right	Left	14	17	2	II	12	+	+	+		94	91	92	100	95	96	100	70	102	73	96	84	17	20	48	41	27
LATL5	28	F	Right	Unknown	12	9	18	I	12	+	+	+		66	83	71	66	83	62	78	72	92	73	80	66	8	20	45	35	69
LATL6	58	M	Right	Ambiguous	12	48	9	I	12	+	+	+		78	102	88	82	103	83	78	70	99	76	95	73	3	20	50	43	56
LATL7	28	F	Right	Left	12	6	21	I	13	+	+	+		95	112	102	88	119	103	92	78	114	85	97	94	14	20	47	40	45
LATL8	27	M	Right	Right	18	6	20	I	9	+	-	-		95	109	101	93	108	94	105	114	119	118	105	116	5	17	49	32	46
LATL9	33	M	Ambiguous	Ambiguous	16	23	9	IV	12	+	+	-		90	94	91	84	95	96	57	69	89	70	105	75	10	20	45	44	65
LATL10	17	M	Right	Left	11	9	7	I	14	+	+	+		89	91	89	86	89	72	107	91	89	88	63	79	3	16	50	36	53
LATL11	37	F	Right	Unknown	16	5	31	III	14	+	+	+		101	117	109	105	116	94	127	122	122	124	97	129	18	20	50	40	60
LATL12	41	F	Right	Unknown	13	27	13	I	14	+	+	+		85	99	91	84	89	74	97	78	111	86	90	88	17	20	50	45	44
LATL13	53	F	Right	Left	15	24	28	I	13	+	+	+		102	103	98	102	103	98	110	78	119	87	96	92	11	20	48	42	31
LATL14	33	M	Right	Unknown	12	18	14	II	13	+	+	+		78	74	74	78	79	74	66	74	74	69	71	59	8	18	46	36	58
HC1	34	F	Ambiguous	N/A	16									N/A	N/A	N/A	N/A	N/A	N/A	N/A	N/A	N/A	N/A	N/A	N/A	18	20	49	43	47
HC2	38	F	Right	N/A	16									N/A	N/A	N/A	N/A	N/A	N/A	N/A	N/A	N/A	N/A	N/A	N/A	17	20	49	43	57
HC3	41	M	Right	N/A	14									N/A	N/A	N/A	N/A	N/A	N/A	N/A	N/A	N/A	N/A	N/A	N/A	17	20	50	43	64
HC4	42	M	Right	N/A	16									N/A	N/A	N/A	N/A	N/A	N/A	N/A	N/A	N/A	N/A	N/A	N/A	18	20	48	35	53
HC5	32	F	Right	N/A	16									N/A	N/A	N/A	N/A	N/A	N/A	N/A	N/A	N/A	N/A	N/A	N/A	18	20	50	44	40
HC6	55	M	Right	N/A	12									N/A	N/A	N/A	N/A	N/A	N/A	N/A	N/A	N/A	N/A	N/A	N/A	13	20	49	35	43
HC7	40	M	Right	N/A	14									N/A	N/A	N/A	N/A	N/A	N/A	N/A	N/A	N/A	N/A	N/A	N/A	17	20	47	32	40
HC8	38	F	Right	N/A	16									N/A	N/A	N/A	N/A	N/A	N/A	N/A	N/A	N/A	N/A	N/A	N/A	18	20	49	44	43
HC9	42	F	Right	N/A	15									N/A	N/A	N/A	N/A	N/A	N/A	N/A	N/A	N/A	N/A	N/A	N/A	18	20	50	35	65
HC10	37	F	Right	N/A	14									N/A	N/A	N/A	N/A	N/A	N/A	N/A	N/A	N/A	N/A	N/A	N/A	17	20	50	45	49
HC11	42	M	Right	N/A	16									N/A	N/A	N/A	N/A	N/A	N/A	N/A	N/A	N/A	N/A	N/A	N/A	14	20	49	43	48
HC12	40	F	Right	N/A	14									N/A	N/A	N/A	N/A	N/A	N/A	N/A	N/A	N/A	N/A	N/A	N/A	17	20	48	40	40
HC13	36	M	Right	N/A	12									N/A	N/A	N/A	N/A	N/A	N/A	N/A	N/A	N/A	N/A	N/A	N/A	16	20	49	35	57
HC14	35	F	Right	N/A	17									N/A	N/A	N/A	N/A	N/A	N/A	N/A	N/A	N/A	N/A	N/A	N/A	16	20	50	44	59
HC15	39	M	Right	N/A	16									N/A	N/A	N/A	N/A	N/A	N/A	N/A	N/A	N/A	N/A	N/A	N/A	18	20	49	37	32
HC16	24	F	Right	N/A	16									N/A	N/A	N/A	N/A	N/A	N/A	N/A	N/A	N/A	N/A	N/A	N/A	18	20	50	45	31
HC17	22	M	Right	N/A	15									N/A	N/A	N/A	N/A	N/A	N/A	N/A	N/A	N/A	N/A	N/A	N/A	18	20	50	44	28
HC18	37	F	Right	N/A	12									N/A	N/A	N/A	N/A	N/A	N/A	N/A	N/A	N/A	N/A	N/A	N/A	17	20	50	44	28
HC19	29	M	Right	N/A	16									N/A	N/A	N/A	N/A	N/A	N/A	N/A	N/A	N/A	N/A	N/A	N/A	18	20	50	42	26
HC20	34	F	Right	N/A	14									N/A	N/A	N/A	N/A	N/A	N/A	N/A	N/A	N/A	N/A	N/A	N/A	16	20	49	32	25
HC21	29	F	Left	N/A	14									N/A	N/A	N/A	N/A	N/A	N/A	N/A	N/A	N/A	N/A	N/A	N/A	18	20	48	41	23

HC22	24	M	Left	N/A	12									N/A	N/A	N/A	N/A	N/A	N/A	N/A	N/A	N/A	N/A	N/A	N/A	14	20	50	43	44
HC23	22	F	Right	N/A	16									N/A	N/A	N/A	N/A	N/A	N/A	N/A	N/A	N/A	N/A	N/A	N/A	18	20	50	41	20
HC24	28	M	Ambiguous	N/A	16									N/A	N/A	N/A	N/A	N/A	N/A	N/A	N/A	N/A	N/A	N/A	N/A	18	20	50	36	28
HC25	29	M	Right	N/A	15									N/A	N/A	N/A	N/A	N/A	N/A	N/A	N/A	N/A	N/A	N/A	N/A	15	20	50	49	24
HC26	45	F	Right	N/A	15									N/A	N/A	N/A	N/A	N/A	N/A	N/A	N/A	N/A	N/A	N/A	N/A	12	19	48	44	34
HC27	23	F	Right	N/A	16									N/A	N/A	N/A	N/A	N/A	N/A	N/A	N/A	N/A	N/A	N/A	N/A	16	20	50	44	28
HC28	33	F	Right	N/A	15									N/A	N/A	N/A	N/A	N/A	N/A	N/A	N/A	N/A	N/A	N/A	N/A	16	20	49	37	25
HC29	33	M	Right	N/A	15									N/A	N/A	N/A	N/A	N/A	N/A	N/A	N/A	N/A	N/A	N/A	N/A	17	20	49	39	22

ATL: Anterior temporal lobectomy; CI: cerebral infarction; FIQ: Full intelligence quotient; HC: healthy controls; LATL: left anterior temporal lobectomy; MFRT: Multiview Face Recognition Test; MST: multiple hippocampal transactions; N/A: not assessed; PI20: the 20-item prosopagnosia index; PIQ: Performance intelligence quotient; PO: Perceptual Organization; PS: Perceptual Speed; RATL: right anterior temporal lobectomy; VC: Verbal Comprehension; VIQ: Verbal intelligence quotient; WAIS-III: Wechsler Adult Intelligence Scale-Third Edition; WM: Working Memory; WMS-R: Wechsler Memory Scale-Revised.
