# Supplementary material for: Facial memory ability and self-awareness in patients with temporal lobe epilepsy after anterior temporal lobectomy
Source: PLoS One. 2021 Apr 1;16(4):e0248785. doi: 10.1371/journal.pone.0248785 (PMC8016293; doi:10.1371/journal.pone.0248785)
Supplement: S3 Table — (PDF) [file pone.0248785.s003.pdf]

S3 **Table. The MFRT results of each participant.**

Subject	MFRT Number of correct answers																														MFRT Accuracy (%)							
	1 picture															3 pictures															1 picture				3 pictures			
	Front view					Oblique view					Noise-masked view					Front view					Oblique view					Noise-masked view					Front view	Oblique view	Noise-masked view	Total	Front view	Oblique view	Noise-masked view	Total
	Interval			Sum	Score	Interval			Sum	Score	Interval			Sum	Score	Interval			Sum	Score	Interval			Sum	Score	Interval			Sum	Score								
	10s (/4)	5s (/4)	0s (/4)	(/12)	(/12.36)	10s (/4)	5s (/4)	0s (/4)	(/12)	(/12.36)	10s (/4)	5s (/4)	0s (/4)	(/12)	(/12.36)	10s (/4)	5s (/4)	0s (/4)	(/12)	(/13.68)	10s (/4)	5s (/4)	0s (/4)	(/12)	(/13.68)	10s (/4)	5s (/4)	0s (/4)	(/12)	(/13.68)								
RATL1	4	4	4	12	12.36	4	4	4	12	12.36	3	4	4	11.0	11.27	2	4	4	10	11.26	0	1	4	5	5.21	1	1	2	4	4.42	100.0	100.0	91.2	97.1	82.3	38.1	32.3	50.9
RATL2	4	4	4	12	12.36	4	3	4	11	11.36	3	4	3	10.0	10.27	2	4	4	10	11.26	2	2	3	7	7.84	2	2	4	8	8.84	100.0	91.9	83.1	91.7	82.3	57.3	64.6	68.1
RATL3	3	4	4	11	11.27	4	4	4	12	12.36	4	4	4	12.0	12.36	2	4	4	10	11.26	2	3	4	9	10.05	1	2	3	6	6.63	91.2	100.0	100.0	97.1	82.3	73.5	48.5	68.1
RATL4	4	4	4	12	12.36	4	4	4	12	12.36	3	4	4	11	11.27	3	2	3	8	9.05	1	3	3	7	7.84	1	2	2	5	5.63	100.0	100.0	91.2	97.1	66.2	57.3	41.2	54.9
RATL5	4	4	4	12	12.36	4	4	4	12	12.36	4	4	4	12	12.36	3	3	4	10	11.26	3	3	2	8	9.26	3	3	3	9	10.26	100.0	100.0	100.0	100.0	82.3	67.7	75.0	75.0
RATL6	3	4	4	11	11.27	3	4	4	11	11.27	3	4	4	11.0	11.27	1	2	4	7	7.63	3	1	4	8	8.84	3	1	4	8	8.84	91.2	91.2	91.2	91.2	55.8	64.6	64.6	61.7
RATL7	3	4	4	11	11.27	3	4	4	11	11.27	4	4	4	12.0	12.36	2	1	4	7	7.63	4	3	3	10	11.47	2	1	4	7	7.63	91.2	91.2	100.0	94.1	55.8	83.8	55.8	65.1
RATL8	4	4	4	12	12.36	2	4	4	10	10.18	4	4	4	12.0	12.36	2	3	3	8	9.05	3	2	3	8	9.05	3	3	3	9	10.26	100.0	82.4	100.0	94.1	66.2	66.2	75.0	69.1
RATL9	3	4	4	11	11.27	4	2	4	10	10.36	3	4	4	11.0	11.27	1	2	4	7	7.63	3	2	3	8	9.05	2	2	3	7	7.84	91.2	83.8	91.2	88.7	55.8	66.2	57.3	59.7
RATL10	4	4	4	12	12.36	4	4	4	12	12.36	4	4	4	12.0	12.36	3	3	4	10	11.26	4	3	3	10	11.47	4	2	4	10	11.26	100.0	100.0	100.0	100.0	82.3	83.8	82.3	82.8
RATL11	4	4	4	12	12.36	3	4	4	11	11.27	4	4	3	11.0	11.36	3	4	4	11	12.47	2	4	4	10	11.26	3	1	1	5	5.84	100.0	91.2	91.9	94.4	91.2	82.3	42.7	72.1
RATL12	4	4	4	12	12.36	4	4	4	12	12.36	4	4	4	12.0	12.36	3	1	4	8	8.84	4	2	3	9	10.26	2	2	4	8	8.84	100.0	100.0	100.0	100.0	64.6	75.0	64.6	68.1
RATL13	3	4	4	11	11.27	2	4	4	10	10.18	3	4	4	11.0	11.27	4	2	4	10	11.26	3	4	2	9	10.47	2	4	2	8	9.26	91.2	82.4	91.2	88.2	82.3	76.5	67.7	75.5
RATL14	2	4	4	10	10.18	3	3	4	10	10.27	3	4	3	10.0	10.27	3	2	4	9	10.05	2	1	4	7	7.63	2	2	3	7	7.84	82.4	83.1	83.1	82.8	73.5	55.8	57.3	62.2
RATL15	3	2	3	8	8.27	2	3	4	9	9.18	4	4	3	11.0	11.36	2	4	4	10	11.26	1	2	4	7	7.63	1	4	2	7	8.05	66.9	74.3	91.9	77.7	82.3	55.8	58.8	65.6
RATL16	4	4	4	12	12.36	3	3	4	10	10.27	4	4	3	11.0	11.36	2	1	2	5	5.63	2	1	3	6	6.63	3	1	1	5	5.84	100.0	83.1	91.9	91.7	41.2	48.5	42.7	44.1
LATL1	2	3	4	9	9.18	1	3	4	8	8.09	4	4	2	10.0	10.36	2	1	3	6	6.63	2	1	3	6	6.63	3	2	3	8	9.05	74.3	65.5	83.8	74.5	48.5	48.5	66.2	54.4
LATL2	4	4	4	12	12.36	4	4	4	12	12.36	4	4	4	12.0	12.36	2	4	4	10	11.26	3	3	3	9	10.26	3	2	4	9	10.05	100.0	100.0	100.0	100.0	82.3	75.0	73.5	76.9
LATL3	3	4	4	11	11.27	3	3	3	9	9.27	2	4	4	10.0	10.18	3	1	3	7	7.84	2	2	1	5	5.84	1	2	3	6	6.63	91.2	75.0	82.4	82.8	57.3	42.7	48.5	49.5
LATL4	4	4	4	12	12.36	3	3	4	10	10.27	3	4	4	11.0	11.27	3	4	3	10	11.47	3	4	4	11	12.47	2	4	4	10	11.26	100.0	83.1	91.2	91.4	83.8	91.2	82.3	85.8
LATL5	3	3	4	10	10.27	2	3	4	9	9.18	4	3	4	11.0	11.36	2	4	4	10	11.26	3	3	3	9	10.26	2	2	4	8	8.84	83.1	74.3	91.9	83.1	82.3	75.0	64.6	74.0
LATL6	4	4	4	12	12.36	3	3	4	10	10.27	4	4	4	12.0	12.36	2	3	3	8	9.05	1	2	3	6	6.63	3	2	2	7	8.05	100.0	83.1	100.0	94.4	66.2	48.5	58.8	57.8
LATL7	4	4	4	12	12.36	4	4	4	12	12.36	4	4	4	12.0	12.36	3	3	4	10	11.26	3	3	3	9	10.26	2	2	4	8	8.84	100.0	100.0	100.0	100.0	82.3	75.0	64.6	74.0
LATL8	4	4	4	12	12.36	3	4	4	11	11.27	3	4	3	10.0	10.27	1	4	2	7	8.05	3	3	2	8	9.26	2	3	3	8	9.05	100.0	91.2	83.1	91.4	58.8	67.7	66.2	64.2
LATL9	3	4	4	11	11.27	4	4	4	12	12.36	4	4	4	12.0	12.36	2	3	3	8	9.05	2	2	4	8	8.84	1	4	4	9	10.05	91.2	100.0	100.0	97.1	66.2	64.6	73.5	68.1
LATL10	4	4	4	12	12.36	4	4	4	12	12.36	4	4	4	12.0	12.36	4	4	2	10	11.68	1	3	3	7	7.84	4	4	4	12	13.68	100.0	100.0	100.0	100.0	85.4	57.3	100.0	80.9
LATL11	4	4	4	12	12.36	4	2	4	10	10.36	3	4	4	11.0	11.27	3	3	4	10	11.26	3	2	3	8	9.05	3	4	3	10	11.47	100.0	83.8	91.2	91.7	82.3	66.2	83.8	77.4
LATL12	4	4	3	11	11.36	4	4	4	12	12.36	4	4	4	12.0	12.36	3	4	3	10	11.47	4	3	3	10	11.47	1	3	4	8	8.84	91.9	100.0	100.0	97.3	83.8	83.8	64.6	77.4
LATL13	3	4	4	11	11.27	4	4	4	12	12.36	3	4	4	11.0	11.27	3	4	4	11	12.47	4	1	3	8	9.05	3	4	2	9	10.47	91.2	100.0	91.2	94.1	91.2	66.2	76.5	77.9
LATL14	3	3	3	9	9.27	1	3	4	8	8.09	4	4	3	11.0	11.36	1	1	3	5	5.42	2	3	3	8	9.05	2	2	2	6	6.84	75.0	65.5	91.9	77.5	39.6	66.2	50.0	51.9
HC1	3	4	4	11	11.27	4	4	4	12	12.36	4	4	4	12.0	12.36	3	4	4	11	12.47	3	2	4	9	10.05	3	4	4	11	12.47	91.2	100.0	100.0	97.1	91.2	73.5	91.2	85.3
HC2	4	4	4	12	12.36	4	4	4	12	12.36	4	4	4	12.0	12.36	3	3	4	10	11.26	1	3	4	8	8.84	4	4	3	11	12.68	100.0	100.0	100.0	100.0	82.3	64.6	92.7	79.9
HC3	4	4	4	12	12.36	3	4	4	11	11.27	4	4	4	12.0	12.36	2	2	4	8	8.84	3	3	4	10	11.26	1	4	3	8	9.05	100.0	91.2	100.0	97.1	64.6	82.3	66.2	71.0
HC4	4	4	4	12	12.36	4	4	4	12	12.36	4	4	4	12.0	12.36	3	4	4	11	12.47	3	3	3	9	10.26	3	1	3	7	7.84	100.0	100.0	100.0	100.0	91.2	75.0	57.3	74.5
HC5	4	3	4	11	11.36	2	4	4	10	10.18	4	4	2	10.0	10.36	2	4	3	9	10.26	2	3	4	9	10.05	1	4	2	7	8.05	91.9	82.4	83.8	86.0	75.0	73.5	58.8	69.1
HC6	4	4	4	12	12.36	3	4	4	11	11.27	4	4	4	12.0	12.36	3	3	4	10	11.26	4	2	4	10	11.26	2	4	4	10	11.26	100.0	91.2	100.0	97.1	82.3	82.3	82.3	82.3
HC7	4	4	4	12	12.36	4	4	4	12	12.36	4	4	4	12.0	12.36	4	4	4	12	13.68	4	2	3	9	10.26	4	2	4	10	11.26	100.0	100.0	100.0	100.0	100.0	75.0	82.3	85.8
HC8	3	4	4	11	11.27	3	4	4	11	11.27	4	4	4	12.0	12.36	2	3	4	9	10.05	1	3	3	7	7.84	2	1	4	7	7.63	91.2	91.2	100.0	94.1	73.5	57.3	55.8	62.2
HC9	4	4	4	12	12.36	4	3	4	11	11.36	4	4	4	12.0	12.36	2	3	4	9	10.05	4	3	4	11	12.47	1	4	4	9	10.05	100.0	91.9	100.0	97.3	73.5	91.2	73.5	79.4
HC10	4	4	4	12	12.36	2	4	4	10	10.18	4	4	4	12.0	12.36	4	4	4	12	13.68	2	3	4	9	10.05	4	4	4	12	13.68	100.0	82.4	100.0	94.1	100.0	73.5	100.0	91.2
HC11	4	4	4	12	12.36	3	4	4	11	11.27	4	4	4	12.0	12.36	4	4	4	12	13.68	3	3	4	10	11.26	4	4	2	10	11.68	100.0	91.2	100.0	97.1	100.0	82.3	85.4	89.2
HC12	3	4	4	11	11.27	3	4	4	11	11.27	3	4	4	11.0	11.27	3	4	3	10	11.47	3	4	4	11	12.47	3	3	4	10	11.26	91.2	91.2	91.2	91.2	83.8	91.2	82.3	85.8
HC13	4	4	4	12	12.36	4	4	4	12	12.36	4	4	4	12.0	12.36	4	4	4	12	13.68	4	3	4	11	12.47	3	4	3	10	11.47	100.0	100.0	100.0	100.0	100.0	91.2	83.8	91.7
HC14	4	4	4	12	12.36	3	4	4	11	11.27	3	4	4	11.0	11.27	4	4	4	12	13.68	2	3	4	9	10.05	4	3	3	10	11.47	100.0	91.2	91.2	94.1	100.0	73.5	83.8	85.8
HC15	4	4	4	12	12.36	4	4	4	12	12.36	4	4	4	12.0	12.36	4	3	4	11	12.47	4	4	3	11	12.68	4	3	4	11	12.47	100.0	100.0	100.0	100.0	91.2	92.7	91.2	91.7
HC16	4	4	4	12	12.36	4	4	4	12	12.36	4	4	4	12.0	12.36	2	4	4	10	11.26	4	4	3	11	12.68	2	4	4	10	11.26	100.0	100.0	100.0	100.0	82.3	92.7	82.3	85.8
HC17	4	4	4	12	12.36	4	4	4	12	12.36	4	4	4	12.0	12.36	3	3	4	10	11.26	3	3	4	10	11.26	3	3	3	9	10.26	100.0	100.0	100.0	100.0	82.3	82.3	75.0	79.9
HC18	4	4	4	12	12.36	4	4	3	11	11.36	4	4	4	12.0	12.36	4	3	4	11	12.47	1	4	4	9	10.05	4	2	3	9	10.26	100.0	91.9	100.0	97.3	91.2	73.5	75.0	79.9
HC19	3	4	3	10	10.27	2	3	4	9	9.18	2	4	4	10.0	10.18	3	1	4	8	8.84	2	2	4	8	8.84	2	1	3	6	6.63	83.1	74.3	82.4	79.9	64.6	64.6	48.5	59.2
HC20	4	4	4	12	12.36	3	4	4	11	11.27	4	4	4	12.0	12.36	3	4	4	11	12.47	3	4	3	10	11.47	3	2	3	8	9.05	100.0	91.2	100.0	97.1	91.2	83.8	66.2	80.4
HC21	3	4	4	11	11.27	3	3	4	10	10.27	2	4	4	10.0	10.18	3	4	4	11	12.47	3	3	4	10	11.26	2	3	3	8	9.05	91.2	83.1	82.4	85.5	91.2	82.3	66.2	79.9
HC22	4	4	4	12	12.36	4	4	4	12	12.36	4	4	4	12.0	12.36	4	4	4	12	13.68	4	4	3	11	12.68	4	4	4	12	13.68	100.0	100.0	100.0	100.0	100.0	92.7	100.0	97.6
HC23	4	3	4	11	11.36	4	4	4	12	12.36	4	4	4	12.0	12.36	4	3	3	10	11.47	3	2	4	9	10.05	2	4	3	9	10.26	91.9	100.0	100.0	97.3	83.8	73.5	75.0	77.4
HC24	4	4	4	12	12.36	3	4	4	11	11.27	4	3	4	11.0	11.36	3	4	4	11	12.47	2	4	4	10	11.26	2	4	4	10	11.26	100.0	91.2	91.9	94.4	91.2	82.3	82.3	85.3
HC25	4	4	4	12	12.36	2	4	4	10	10.18	4	4	4	12.0	12.36	1	3	4	8	8.84	2	3	2	7	8.05	2	2	4	8	8.84	100.0	82.4	100.0	94.1	64.6	58.8	64.6	62.7
HC26	4	4	4	12	12.36	4	4	4	12	12.36	4	4	4	12.0	12.36	4	4	4	12	13.68	2	2	3	7	7.84	3	3	3	9	10.26	100.0	100.0	100.0	100.0	100.0	57.3	75.0	77.4
HC27	3	3	4	10	10.27	3	4	3	10	10.27	3	4	4	11.0	11.27	2	3	4	9	10.05	0	3	3	6	6.63	1	1	3	5	5.42	83.1	83.1	91.2	85.8	73.5	48.5	39.6	53.8
HC28	3	4	4	11	11.27	2	3	4	9	9.18	3	4	4	11.0	11.27	1	3	3	7	7.84	3	2	1	6	7.05	1	2	4	7	7.63	91.2	74.3	91.2	85.5	57.3	51.5	55.8	54.9
HC29	4	4	4	12	12.36	4	4	3	11	11.36	4	4	4	12.0	12.36	4	3	3	10	11.47	3	4	4	11	12.47	3	2	3	8	9.05	100.0	91.9	100.0	97.3	83.8	91.2	66.2	80.4

HC: healthy controls; LATL: left anterior temporal lobectomy; MFRT: Multiview Face Recognition Test; N/A: not assessed; RATL: right anterior temporal lobectomy.
